# Neural dynamics during the vocalization of ‘uh’ or ‘um’

**DOI:** 10.1038/s41598-020-68606-x

**Published:** 2020-07-20

**Authors:** Ayaka Sugiura, Zahraa Alqatan, Yasuo Nakai, Toshimune Kambara, Brian H. Silverstein, Eishi Asano

**Affiliations:** 1 Department of Pediatrics, Wayne State University, Detroit, MI 48201 USA; 2 Department of Neurology, Wayne State University, Detroit, MI 48201 USA; 3 School of Medicine, Wayne State University, Detroit, MI 48201 USA; 4 Translational Neuroscience Program, Wayne State University, Detroit, MI 48201 USA; 5 Department of Neurodiagnostics, Children’s Hospital of Michigan, Wayne State University, 3901 Beaubien St, Detroit, MI 48201 USA; 6 Division of Pediatric Neurology, Children’s Hospital of Michigan, Detroit, MI 48201 USA; 7 Department of Neurological Surgery, Wakayama Medical University, Wakayama, 6418509 Japan; 8 Department of Psychology, Hiroshima University, Hiroshima, 7398524 Japan

**Keywords:** Neuroscience, Neurology

## Abstract

People occasionally use filler phrases or pauses, such as “uh”, “um”, or “y’know,” that interrupt the flow of a sentence and fill silent moments between ordinary (non-filler) phrases. It remains unknown which brain networks are engaged during the utterance of fillers. We addressed this question by quantifying event-related cortical high gamma activity at 70–110 Hz. During extraoperative electrocorticography recordings performed as part of the presurgical evaluation, patients with drug-resistant focal epilepsy were instructed to overtly explain, in a sentence, ‘*what is in the image* (subject)’, ‘*doing what* (verb)’, ‘*where* (location)’, and ‘*when* (time)’. Time–frequency analysis revealed that the utterance of fillers, compared to that of ordinary words, was associated with a greater magnitude of high gamma augmentation in association and visual cortex of either hemisphere. Our preliminary results raise the hypothesis that filler utterance would often occur when large-scale networks across the association and visual cortex are engaged in cognitive processing, including lexical retrieval as well as verbal working memory and visual scene scanning.

## Introduction

Regardless of age, gender, or native language, healthy individuals use filler phrases, also known as filled pauses, during spontaneous speech^1^. Frequent utterance of fillers is tightly associated with increased effort to recall or search for a relevant word^2^, increased anxiety^3^, and divided attention^4^. Disfluent non-native speakers compared to native ones as well as dysphasic patients compared to non-dysphasic ones more frequently utter fillers during verbal communication^5,6^. Practice and preparation are effective methods to reduce the rate of filler utterance during interviews or presentations because the word recall process becomes more automatic and less effortful^7^.

*What happens in the cerebral cortex when one utters a filler?* Only a small number of studies have attempted to determine the neural correlates of filler utterances. Effective study design is a consistent challenge in the field due to the unpredictable timing of naturally occurring filler phrases or pauses. In a study of six healthy adults using functional MRI (fMRI)^8^, participants were instructed to speak whatever came to mind when viewing Rorschach inkblot plates. The authors reported that trials accompanied by overt filler pauses, compared to those accompanied by complete silent pauses, was associated with increased hemodynamic activation in the left superior temporal gyrus. Another fMRI study characterized the spatial pattern of hemodynamic activation when participants listened to other’s speeches including fillers to determine the neural correlates of *listening* and not *utterance* of fillers^9^.

Measurement of event-related high gamma activity on electrocorticography (ECoG), a presurgical evaluation method for patients with drug-resistant epilepsy^10^, provides a unique opportunity to quantify the rapid dynamics of human perception and cognition without increasing the risk of surgical complications^11^. Task-related high gamma activity at 70–110 Hz is a summary measure of local cortical engagement with a temporal resolution of tens of milliseconds^12^. Augmentation of high gamma amplitude has been reported to be tightly associated with an increase in firing rate^13^, hemodynamic activation^14^, glucose metabolism^15^, and the probability of stimulation-induced functional impairment^16^. Conversely, attenuation of high gamma amplitude is associated with a reduced firing rate and hemodynamic deactivation^17^. Because of its outstanding signal fidelity, ECoG recording is suggested to be capable of accurately measuring the spatiotemporal dynamics of event-related neural modulations at a single-trial level in an individual patient^18,19^.

**Figure 1:**
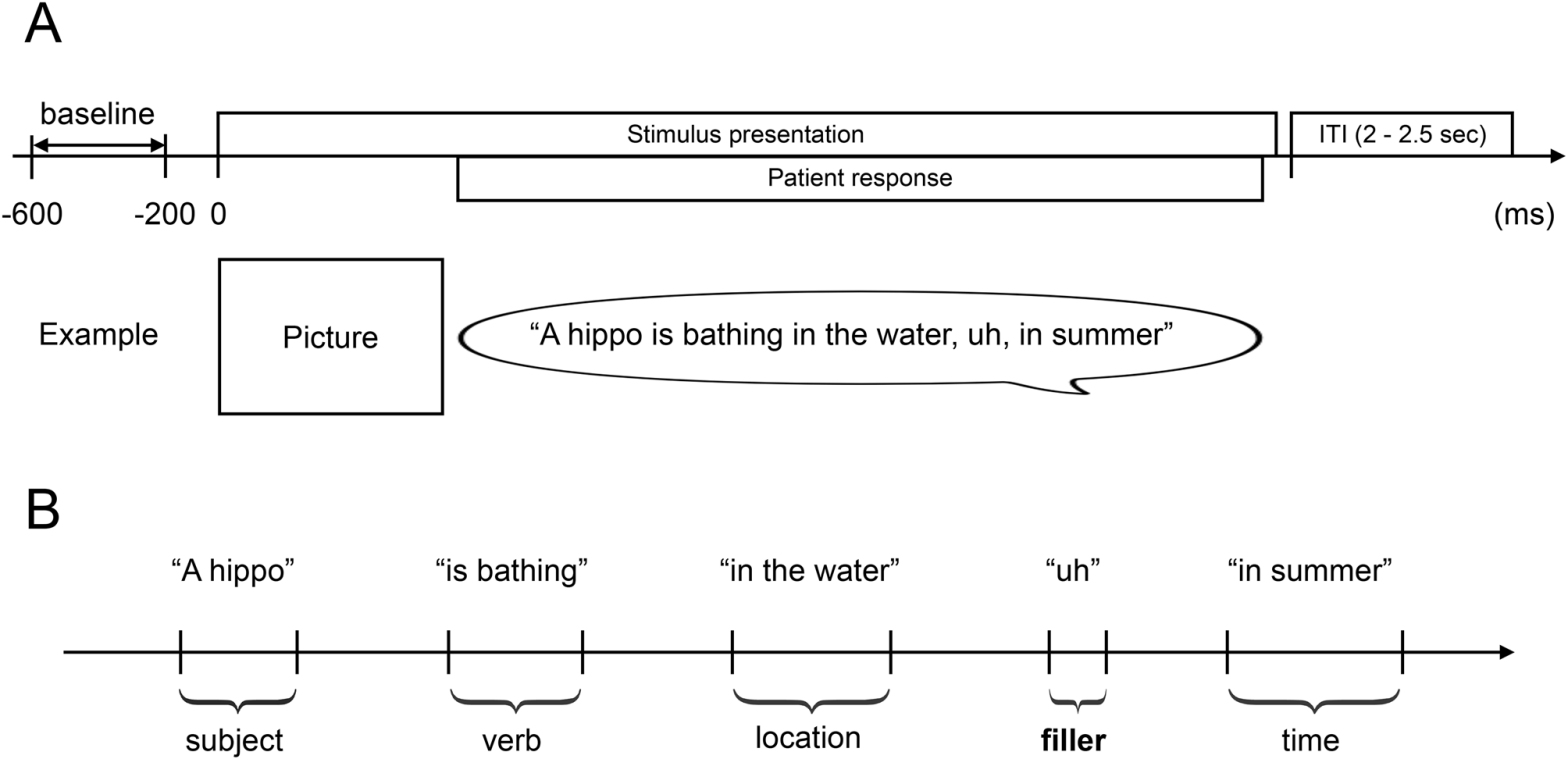
Sentence production task. (**A**) Timeline of the task. (**B**) Event classification. Each patient was instructed to look at and overtly explain a visual scene in a sentence, including the subject, verb, location, and time in any order. At the end of each response, the examiner pressed a button to present the next photograph following the presentation of a fixation cross in the center of the screen for 2 or 2.5 s. All phrases were classified as either filler or non-filler.

In the present study, during extraoperative ECoG recording, each participant was instructed to overtly explain the content of a given image with a sentence including the subject (e.g., *A baby*), verb (*plays with a dog*), location (*at the beach*), and time (*during the day;* Fig. 1). Due to the challenging nature of this task, all participants intermittently used fillers during sentence production. Although we did not originally employ this task to study the neural correlates of filler utterances versus ordinary phrases, it provided a rare opportunity to determine the spatiotemporal characteristics of high gamma augmentation during this ubiquitous, yet unpredictable, human behavior.

Given that filler phrases are associated with recall effort and word search^2^, we hypothesized that the spontaneous utterance of fillers, compared to that of non-filler words, would be associated with greater high gamma augmentation across large-scale networks of the association cortex. This hypothesis was further motivated by previous imaging studies of healthy children and adults, which reported that these regions were activated, to the largest extent, during tasks requiring the selection of optimal words among competing alternatives^20–25^. Furthermore, studies of patients with stroke and primary progressive aphasia suggest an association between more severe damage to the association cortex of the left hemisphere and increased rate of filler utterance due to the loss of word retrieval ability^3,26–29^.

## Methods

### Participants

We studied three native English-speaking patients (Table 1; age: 15, 16, and 17 years; 1 female), who underwent the sentence production task (Fig. 1A) during extraoperative subdural ECoG recording at Children’s Hospital of Michigan, Detroit, USA. None of these patients had massive brain malformations observable on an MRI or severe cognitive impairment defined by a verbal IQ of < 70. This study, approved by the Institutional Review Board at Wayne State University, was performed in accordance with the approved guidelines. Informed consent and assent were obtained from the guardians of patients and patients, respectively.

**Table 1:** Patient profile.

	Patient		
	1	2	3
Age (years)	15	17	16
Sex	Male	Male	Female
Sampled hemisphere	Left	Both	Both
Handedness	Right	Right	Right
Estimated epileptogenic zone	Left temporal	Left frontal	Right frontal
Antiepileptic drug	LCM, CLB	CLB	TPM, CLB
MRI	Nonlesional	Nonlesional	Nonlesional
PPVT	91	102	130

*CLB* clobazam, *LCM* lacosamide, *TPM* topiramate, *PPVT* Peabody picture vocabulary test. Because of the right-handedness and absence of early neocortical lesions in the left hemisphere, all patients were assumed to have left-hemispheric language dominance^30^. Electrical stimulation mapping indeed localized the essential language areas in the left superior-temporal and inferior-frontal gyri of Patients 1 and 2, who were suspected of having the epileptogenic zone in the left hemisphere. Conversely, electrical stimulation mapping of the right hemisphere did not elicit language symptoms in Patient 3.

### Acquisition of ECoG and three-dimensional magnetic resonance surface images

**Figure 2:**
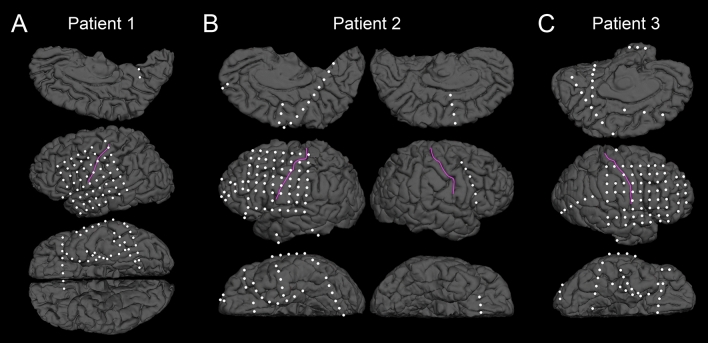
Location of subdural electrodes included in the analysis. (**A**) Patient 1. (**B**) Patient 2. (**C**) Patient 3. The pink line delineates the central sulcus.

ECoG and MRI data acquisition methods were described elsewhere^30^. Platinum disk electrodes (10 mm center-to-center distance; 3 mm exposed diameter) were placed in the subdural space of the hemisphere estimated to contain the epileptogenic zone, based on collective evidence from the noninvasive presurgical evaluation^31^. ECoG signals were continuously acquired at a sampling rate of 1,000 Hz using the Nihon Kohden Neurofax 1100A Digital System (Nihon Kohden America Inc., Foothill Ranch, CA, USA). Channels classified as seizure onset zone, those generating interictal spike discharges, as well as those showing artifacts during the task were excluded from further analysis. This is a common procedure across ECoG studies of event-related high gamma activity and expected to improve the generalizability of the findings^12,32–34^. The number of nonepileptic channels included in the analysis ranged from 100 to 128 per patient. We created a three-dimensional surface image for each patient with electrode sites defined directly on the pial surface^30^. FreeSurfer scripts were used to parcellate the cortical gyri of each individual surface image (https://surfer.nmr.mgh.harvard.edu), in order to determine the anatomical label of each electrode location ^35,36^ (Fig. 2). All three patients had electrode coverages commonly involving the lateral frontal, parietal, and temporal regions.

### Sentence production task

At the bedside during extraoperative ECoG recording, participants were instructed to freely explain, in a sentence, the content of a visual scene. Each scene was a photograph sampled from the International Affective Picture System^37^. Each photograph was 9 × 12 cm and presented at the center of a 19 inch LCD monitor placed 60 cm in front of the patient. Participants were instructed to include the following domains in the sentence in any order: ‘subject (e.g., *A hippo*)’, ‘verb (*is bathing*)’, ‘location (*in the water*)’, and ‘time (*in summer*)’ (Fig. 1A). Each participant was instructed to say, ‘I don’t know’, in case she/he failed to understand the content of a given scene. Each trial began with a 2.0 or 2.5 s fixation cross followed by the presentation of the photograph. The scene was presented until the patient completed their response, at which point the examiner manually started the next trial. Overt verbal responses were recorded using a WS-823 digital voice recorder (Olympus America Inc, Hauppauge, NY, USA) and synchronized with ECoG signals via a DC input to the ECoG amplifier^10^. The timing of the picture stimulus presentation was likewise synchronized using a photosensor attached to the corner of the LCD monitor and the ECoG amplifier via DC input.

### Event classification and marking

The onset and offset of filler and ordinary phrases were identified and marked using recorded vocal sounds synchronized to the ECoG signal (Fig. 1B). Fillers were defined as an extraneous word or set of words (e.g., “uh”, “um”, “y’know”, or “well”^1,38^). We used Cool Edit Pro version 2 (Syntrillium Software Corp., Phoenix, AZ, USA) to aid in the manual marking of each phrase of interest^39^.

### Time–frequency analysis

We determined the dynamics of high gamma modulations during filler and non-filler utterances using a method similar to what we have previously reported^30^. Briefly, we applied a complex demodulation method to transform ECoG signals from the time–voltage into time–frequency domain in steps of 5 Hz and 10 ms^40,41^. For each ECoG channel, we quantified the mean percentage change of high gamma amplitude within 70–110 Hz in 10 ms bins relative to a 400-ms reference period at 600–200 ms prior to the presentation of the photograph stimulus (Fig. 1). High gamma amplitude, time-locked to utterance onset and offset, was plotted as a function of time (Figs. 4, S1).

### Statistical analysis to determine the effect of filler utterances on high gamma activity

**Figure 3:**
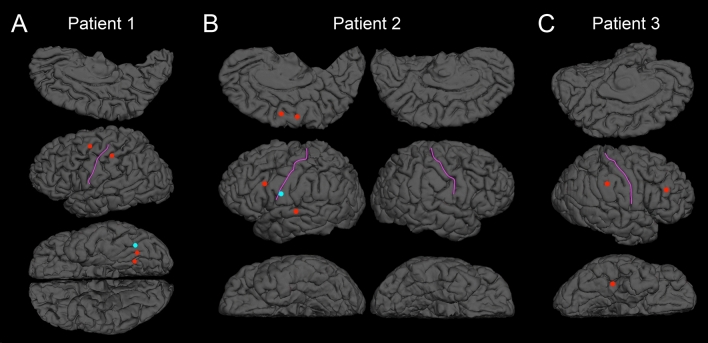
Spatial characteristics of filler-preferential high gamma augmentation and attenuation. (**A**) Patient 1. (**B**) Patient 2. (**C**) Patient 3. All electrode sites that showed significant filler-preferential high gamma augmentation (red circles) or attenuation (blue circles) based on the mixed model analysis. Filler-preference electrodes were defined as having a ‘filler utterance’ effect on high gamma activity (*t*-score) that was either above or below two standard deviations from the mean across all electrodes in given patients. The pink line delineates the central sulcus.

To determine whether fillers accounted for the variance in utterance-related high gamma modulations, we employed a mixed model analysis at each electrode site of a given patient (SPSS Statistics 25, IBM Corp., Chicago, IL, USA). The dependent variable was the percent change of high gamma activity during a 300 ms utterance period. The following variables were treated as fixed effects: (1) ‘filler utterance’ (1 if an uttered phrase was a filler and 0 if a non-filler), (2) ‘onset/offset of phrase’ (1 during the 300 ms period immediately after utterance onset and 0 during the 300 ms period immediately before utterance offset), (3) trial number, and (4) phrase duration (ms). This analysis was designed to determine whether a filler was associated with increased neural activation independently of the three co-variables mentioned above. The intercept was treated as a random effect. The statistical significance threshold was set at *p* = 0.05. Cortical regions with preferential activation during fillers were identified as those with high gamma effects exceeding two standard deviations above or below the mean across all electrodes for the patient (Fig. 3).

## Results

**Figure 4:**
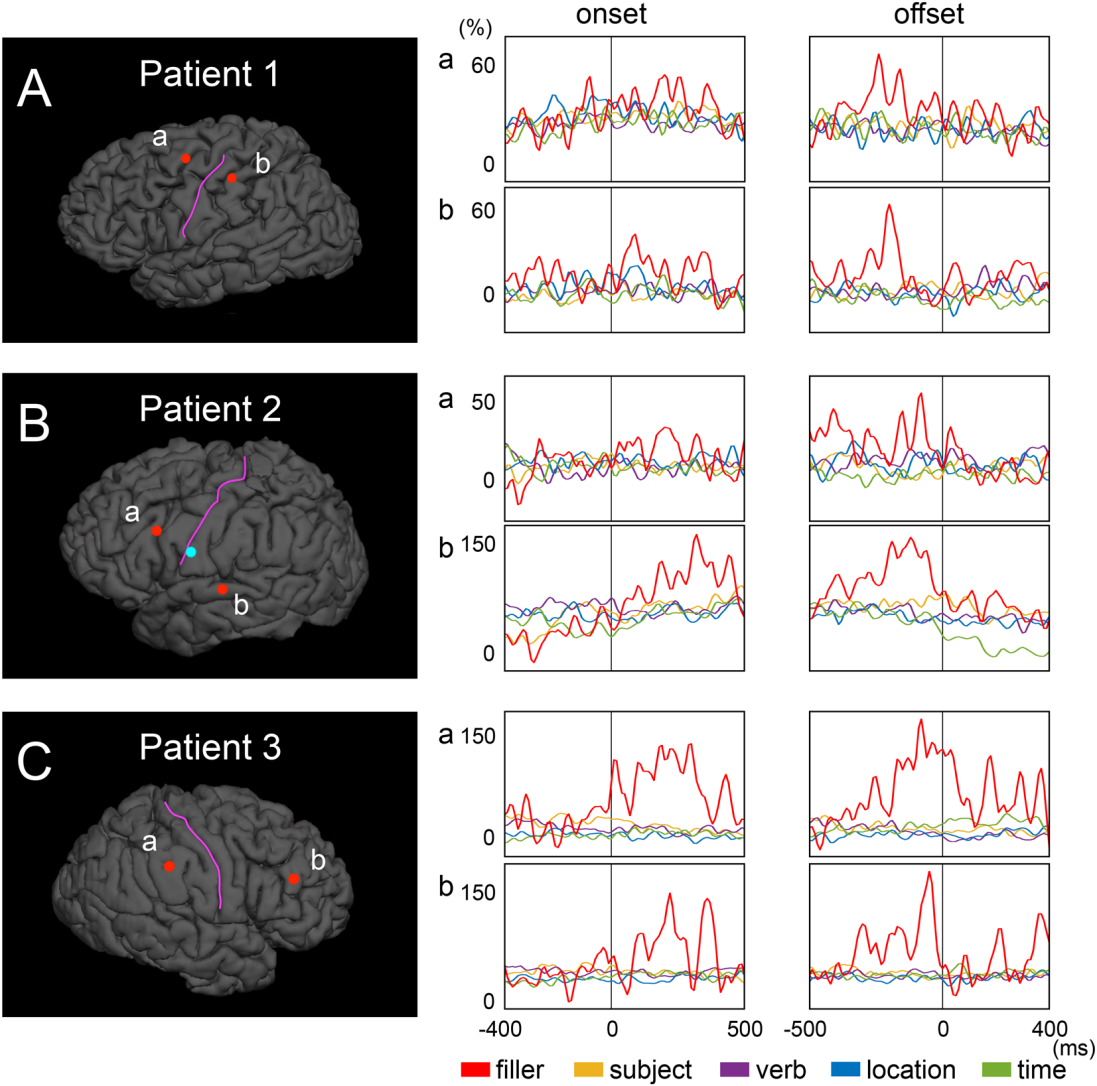
Temporal dynamics of utterance-related high gamma augmentation. The temporal dynamics of high gamma amplitude (% change) in (**A**) Patient 1, (**B**) Patient 2, and (**C**) Patient 3. The mixed model analysis showed significant filler-preferential high gamma augmentation in these electrode sites.

Table 2 summarizes the behavioral data of given patients, including the number and duration of filler and non-filler utterance. The duration of filler utterances was shorter than that of the utterance of ordinary phrases (Table 2). Figure 3 presents the locations of electrode sites at which the filler effect was above or below two standard deviations from the mean across all electrode sites for a given patient. Ten sites in the association cortex and one in the left lingual gyrus (i.e., visual cortex) showed filler-preferential high gamma augmentation. Figure 4 shows the temporal dynamics of utterance-related high gamma activity at sites showing filler-preferential high gamma augmentation. Blue circles (N = 2) in Fig. 3 indicate the locations of sites showing filler-preferential high gamma attenuation. Table 3 summarizes the mixed model coefficients, *t*-scores, and confidence intervals of the filler effects at the 13 sites mentioned above. Online Supplementary Figure S1shows utterance-related high gamma augmentation at a face sensorimotor cortical site taking place commonly during filler and non-filler utterance.

**Table 2:** Behavioral data.

	Patient		
	1	2	3
Number of trials	77	96	94
Number of filler phrases	21	16	3
**Duration of utterance(mean ± SE/median) (ms)**			
Filler	392.7 ± 36.4/359	471.31 ± 66.7/403.5	401.3 ± 74.0/422
Subject	962.5 ± 83.4/781	634.91 ± 38.6/530	525.8 ± 25.9 /457
Verb	2,431.2 ± 160.1/1988	1,075.6 ± 66.4/936	1,133.9 ± 54.9/1,122.5
	1503.3 ± 174.0/1,081	839.7 ± 44.2/ 681	958.4 ± 54.8/820
Time	1,482.8 ± 144.6/1,073.5	634.9 ± 31.0/530	869.4 ± 34.7/805

*SE* standard error.

**Table 3:** Results of mixed model analysis to assess the filler effect on high gamma activity.

Patient	Hemisphere	Anatomical location	Mixed model coefficient	95% confidence interval		*t*-score	*p*-value
				Lower limit	Upper limit		
1	Left	Lingual	0.264	0.136	0.391	4.065	0.0001*
1	Left	Supramarginal	0.134	0.058	0.209	3.49	0.0005*
1	Left	Caudal middle frontal	0.14	0.056	0.224	3.267	0.0011*
1	Left	Fusiform	0.123	0.042	0.204	2.981	0.003
1	Left	Inferior temporal	− 0.168	− 0.279	− 0.057	− 2.982	0.003
2	Left	Superior temporal	0.35	0.19	0.51	4.293	< 0.0001*
2	Left	Superior frontal	0.19	0.081	0.299	3.435	0.0006*
2	Left	Superior frontal	0.138	0.035	0.241	2.64	0.0085
2	Left	Pars opercularis of inferior frontal gyrus	0.138	0.034	0.242	2.611	0.0092
2	Left	Postcentral	− 0.323	− 0.47	− 0.177	− 4.324	< 0.0001*
3	Right	Fusiform	0.377	0.188	0.567	3.908	0.0001*
3	Right	Supramarginal	0.791	0.376	1.206	3.745	0.0002*
3	Right	Pars triangularis of inferior frontal gyrus	0.423	0.097	0.749	2.547	0.0111

*p values survived the FDR correction for multiple comparisons within a given patient.

## Discussion

### Significance of filler-preferential high gamma augmentation

The present study indicated that the utterance of fillers, compared to that of ordinary ones, was associated with greater high gamma augmentation primarily in the association cortex. A plausible explanation for our ECoG observation is that filler utterances are more likely to occur while large-scale networks across the association cortex remain engaged in cognitive processing prior to motor responses (i.e., verbal articulation). This hypothesis is consistent with the generally accepted notion that filler utterances are a behavioral marker of increased effort to recall, search, or select a relevant word^2^. The involvement of large-scale association networks reflects the complexity of the sentence production task. To observe and fully describe a pictured scene involves integrating perceptual, working memory, motor, and cognitive functions at least including the semantic processing of the perceived image as well as lexical and phonological access in a sentence context^42,43^. The sentence production task requires extensive analysis of the visual scene involving multiple domains and a long duration of utterance response. Collective evidence indicates that semantic, lexical, and phonological processes are exerted by large-scale networks in the temporal, parietal, and frontal lobe association cortex with left-hemispheric dominance^33,44,45^. Linking each part of the description into a single sentence also requires substantial verbal working memory activation, which may further involve the association cortex of either hemisphere^46–48^. In contrast, overt production of non-filler phrases was previously reported to maximize the degree of neural activation in the primary sensorimotor cortex following the subsidence of neural activation in the left inferior frontal gyrus^32,44^.

One cannot rule out the possibility that our patients spontaneously used fillers as a method to communicate their intention^49,50^. In other words, one may subconsciously use fillers as a signal to infer that she/he still intends to speak further or show a need for time to collect thoughts. A behavioral study previously reported that the audience rated speakers using filler pauses higher in presentation skills than those using complete silent pauses^51^. A previous fMRI study of 16 healthy adults investigated the effect of *listening* to speech including fillers^9^; thereby, participants were instructed to listen to auditory sentences delivered via headphones carefully. This fMRI study reported that speech including fillers, compared to fluent speech, elicited greater degrees of hemodynamic activation in the superior temporal gyri as well as medial frontal regions.

The present study did not provide the causal evidence suggesting that filler utterance indeed facilitated the cognitive process. Our observation of filler-preferential neuronal activation in the association cortex does not indicate that frequent usage of fillers improves the verbal response.

Since all patients were adolescents, we cannot rule out the possibility that the reported neuronal dynamics could be specific to this phase of development.

### Methodological considerations

The small sample size is a major limitation of the present study. Thus, one should consider this research as a hypothesis-generating study rather than as a definitive investigation. However, because the signal fidelity of ECoG is more than 100 times better than that of scalp EEG^19^, a number of studies suggest that one can evaluate task-related high gamma modulations on a per-trial basis^12,18,52^. Each patient uttered filler phrases only three to 21 times but more than 300 ordinary phrases during the task (Table 2). Such small numbers of filler utterance limited the statistical power in the mixed model analysis. Only seven of the 11 sites showing a positive filler effect on high gamma activity would survive the FDR correction for approximately 100 subdural electrode channels per patient (Table 3). Correction for multiple tests decreases the risk of Type I error but increases the risk of Type II error; given the exploratory nature of this analysis, we opted not to apply the FDR correction. Further studies using a larger number of patients and trials are necessary to validate or disprove the hypothesis generated in the present study. For example, analysis of ECoG signals during task-free communications may increase the chance of securing sufficient statistical power^53^.

In the present study, we computed the percentage change of high gamma amplitude relative to that during a reference period. This analytic approach was based on the assumption that the patient was resting during the reference period between trials.

## Supplementary information

Supplementary information.

## References

[1] Laserna CM, Seih Y-T, Pennebaker JW (2014). Um.. who like says you know. J. Lang. Soc. Psychol..

[2] Dockrell JE, Messer D, George R, Wilson G (1998). Children with word-finding difficulties–prevalence, presentation and naming problems. Int J Lang Commun Disord.

[3] Christenfeld N, Creager B (1996). Anxiety, alcohol, aphasia, and ums. JPSP.

[4] Oomen CCE, Postma A (2001). Effects of divided attention on the production of filled pauses and repetitions. J. Speech. Lang. Hear. Res..

[5] Norris MR, Drummond SS (1998). Communicative functions of laughter in aphasia. J. Neurolinguist..

[6] Tomokiyo, L. M. *Linguistic properties of non-native speech. in 3*, 1335–1338 (IEEE, 2000).

[7] Saul, M. *Caroline Kennedy no whiz with words. New York Daily News*. https://www.nydailynews.com/news/politics/caroline-kennedy-no-whiz-words-article-1.355586. Accessed 3 July 2020 (2008).

[8] Matsumoto K (2013). Frequency and neural correlates of pauses in patients with formal thought disorder. Front. Psychiatry.

[9] Eklund, R. & Ingvar, M. *Supplementary Motor Area Activation in Disfluency Perception: An fMRI Study of Listener Neural Responses to Spontaneously Produced Unfilled and Filled Pauses. in 2016*, 1378–1381 (ISCA, 2016).

[10] Kambara T, Brown EC, Silverstein BH, Nakai Y, Asano E (2018). Neural dynamics of verbal working memory in auditory description naming. Sci. Rep..

[11] Uematsu M, Matsuzaki N, Brown EC, Kojima K, Asano E (2013). Human occipital cortices differentially exert saccadic suppression: intracranial recording in children. NeuroImage.

[12] Crone NE, Korzeniewska A, Franaszczuk PJ (2011). Cortical gamma responses: searching high and low. Int. J. Psychophysiol..

[13] Ray S, Crone NE, Niebur E, Franaszczuk PJ, Hsiao SS (2008). Neural correlates of high-gamma oscillations (60–200 Hz) in macaque local field potentials and their potential implications in electrocorticography. J. Neurosci..

[14] Scheeringa R (2011). Neuronal dynamics underlying high- and low- frequency EEG oscillations contribute independently to the human BOLD signal. Neuron.

[15] Nishida M, Juhász C, Sood S, Chugani HT, Asano E (2008). Cortical glucose metabolism positively correlates with gamma-oscillations in nonlesional focal epilepsy. NeuroImage.

[16] Arya R, Horn PS, Crone NE (2018). ECoG high-gamma modulation versus electrical stimulation for presurgical language mapping. Epilepsy Behav..

[17] Shmuel A, Augath M, Oeltermann A, Logothetis NK (2006). Negative functional MRI response correlates with decreases in neuronal activity in monkey visual area V1. Nat. Neurosci..

[18] Flinker A (2010). Single-trial speech suppression of auditory cortex activity in humans. J. Neurosci..

[19] Ball T, Kern M, Mutschler I, Aertsen A, Schulze-Bonhage A (2009). Signal quality of simultaneously recorded invasive and non-invasive EEG. NeuroImage.

[20] Thompson-Schill SL, D'Esposito M, Kan IP (1999). Effects of repetition and competition on activity in left prefrontal cortex during word generation. Neuron.

[21] Crosson B (2001). Relative shift in activity from medial to lateral frontal cortex during internally versus externally guided word generation. J. Cognit. Neurosci..

[22] Holland SK (2001). Normal fMRI brain activation patterns in children performing a verb generation task. NeuroImage.

[23] Costafreda SG (2006). A systematic review and quantitative appraisal of fMRI studies of verbal fluency: role of the left inferior frontal gyrus. Hum. Brain Mapp..

[24] Grèzes J, Decety J (2001). Functional anatomy of execution, mental simulation, observation, and verb generation of actions: a meta-analysis. Hum. Brain Mapp..

[25] Wagner S, Sebastian A, Lieb K, Tüscher O, Tadić A (2014). A coordinate-based ALE functional MRI meta-analysis of brain activation during verbal fluency tasks in healthy control subjects. BMC Neurosci..

[26] Love T, Swinney D, Wong E, Buxton R (2002). Perfusion imaging and stroke: a more sensitive measure of the brain bases of cognitive deficits. Aphasiology.

[27] Wilson SM (2010). Connected speech production in three variants of primary progressive aphasia. Brain.

[28] Mack JE (2015). What do pauses in narrative production reveal about the nature of word retrieval deficits in PPA?. Neuropsychologia.

[29] Thothathiri M, Schwartz MF, Thompson-Schill SL (2010). Selection for position: the role of left ventrolateral prefrontal cortex in sequencing language. Brain Lang..

[30] Nakai Y (2017). Three- and four-dimensional mapping of speech and language in patients with epilepsy. Brain.

[31] Asano E, Juhász C, Shah A, Sood S, Chugani HT (2009). Role of subdural electrocorticography in prediction of long-term seizure outcome in epilepsy surgery. Brain.

[32] Flinker A (2015). Redefining the role of Broca's area in speech. Proc. Natl. Acad. Sci. U.S.A..

[33] Forseth KJ (2018). A lexical semantic hub for heteromodal naming in middle fusiform gyrus. Brain.

[34] Kambara T (2018). Presurgical language mapping using event-related high-gamma activity: The Detroit procedure. Clin. Neurophysiol..

[35] Desikan RS (2006). An automated labeling system for subdividing the human cerebral cortex on MRI scans into gyral based regions of interest. NeuroImage.

[36] Nishida M (2017). Brain network dynamics in the human articulatory loop. Clin. Neurophysiol..

[37] Lang, P. J., Bradley, M. M. & Cuthbert, B. N. *International affective picture system (IAPS): affective ratings of pictures and instruction manual. Technical Report A-8* (2008).

[38] Bortfeld H, Leon SD, Bloom JE, Schober MF, Brennan SE (2001). Disfluency rates in conversation: effects of age, relationship, topic, role, and gender. Lang. Speech.

[39] Brown EC (2008). In vivo animation of auditory-language-induced gamma-oscillations in children with intractable focal epilepsy. NeuroImage.

[40] Papp N, Ktonas P (1977). Critical evaluation of complex demodulation techniques for the quantification of bioelectrical activity. Biomed. Sci. Instrum..

[41] Hoechstetter K (2004). BESA source coherence: a new method to study cortical oscillatory coupling. Brain Topogr..

[42] Shelton JR, Caramazza A (1999). Deficits in lexical and semantic processing: implications for models of normal language. Psychon. Bull. Rev..

[43] Dell GS, O'Seaghdha PG (1992). Stages of lexical access in language production. Cognition.

[44] Nakai Y (2019). Four-dimensional functional cortical maps of visual and auditory language: Intracranial recording. Epilepsia.

[45] Hamberger MJ, Habeck CG, Pantazatos SP, Williams AC, Hirsch J (2014). Shared space, separate processes: Neural activation patterns for auditory description and visual object naming in healthy adults. Hum. Brain Mapp..

[46] Kambara T (2017). Spatio-temporal dynamics of working memory maintenance and scanning of verbal information. Clin. Neurophysiol..

[47] Chen SHA, Desmond JE (2005). Cerebrocerebellar networks during articulatory rehearsal and verbal working memory tasks. NeuroImage.

[48] Paulesu E, Frith CD, Frackowiak RSJ (1993). The neural correlates of the verbal component of working memory. Nature.

[49] Fox Tree JE (2007). Folk notions of um and uh, you know, and like. Text Talk Interdiscip. J. Lang. Discourse Commun. Stud..

[50] Maclay H, Osgood CE (1959). Hesitation phenomena in spontaneous english speech. WORD.

[51] Christenfeld N (1995). Does it hurt to say um?. J. Nonverbal Behav..

[52] Johnson EL, Tang L, Yin Q, Asano E, Ofen N (2018). Direct brain recordings reveal prefrontal cortex dynamics of memory development. Sci. Adv..

[53] Arya R (2015). Electrocorticographic language mapping in children by high-gamma synchronization during spontaneous conversation: Comparison with conventional electrical cortical stimulation. Epilepsy Res..

