# Supplementary material for: Neural dynamics during the vocalization of ‘uh’ or ‘um’
Source: Sci Rep. 2020 Jul 20;10:11987. doi: 10.1038/s41598-020-68606-x (PMC7371885; doi:10.1038/s41598-020-68606-x)
Supplement: Supplementary file 1 — Supplementary information. [file 41598_2020_68606_MOESM1_ESM.docx]

**Supplementary document**

**in**

**Neural dynamics during the vocalization of ‘uh’ or ‘um’.**

Ayaka Sugiura ; Zahraa Alqatan ; Yasuo Nakai ; Toshimune Kambara ;

Brian H. Silverstein ; Eishi Asano

**Supplmentary Figure S1. Utterance-related high-gamma augmentation at a primary sensorimotor site.**

**
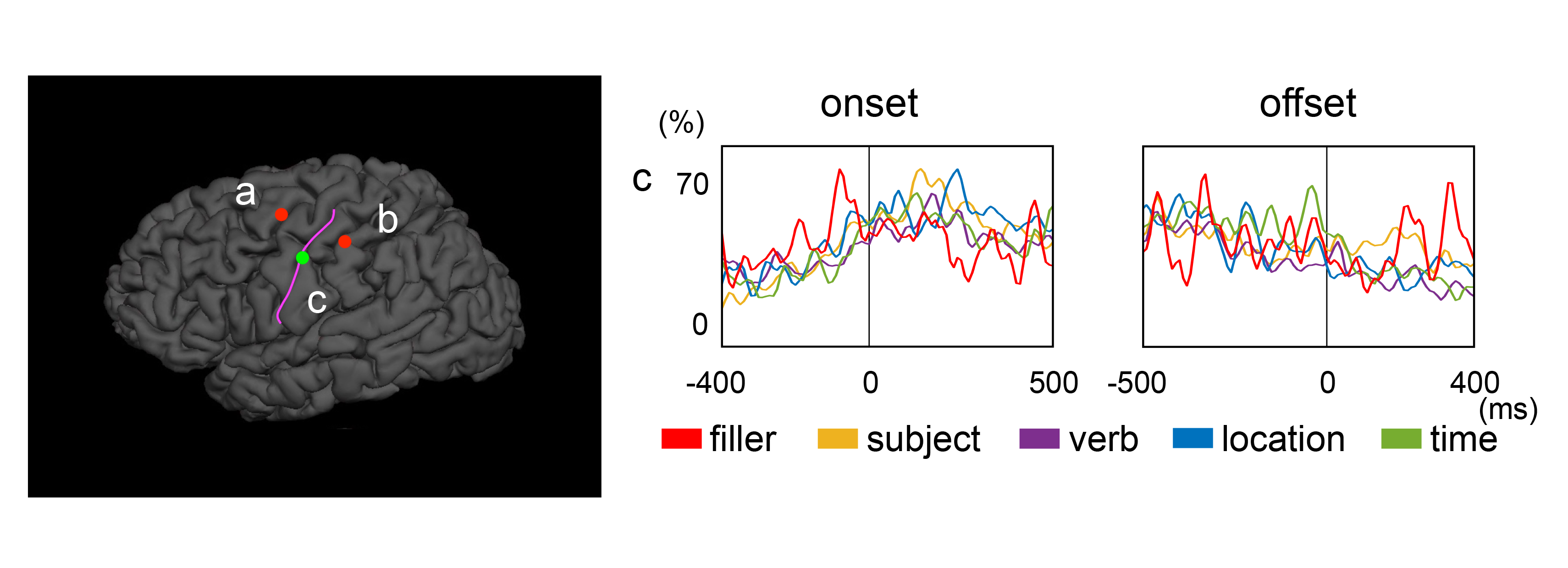
**

The temporal dynamics of high-gamma amplitude (% change) at a primary sensorimotor cortex site (Channel C) in Patient 1 is presented.
